# *Marine Drugs* Best Paper Award 2013

**DOI:** 10.3390/md11030581

**Published:** 2013-02-26

**Authors:** Ophelia Han

**Affiliations:** MDPI AG, Postfach, CH-4005 Basel, Switzerland and MDPI Branch Office, Beijing, China; E-Mail: ophelia.han@mdpi.com

To recognize the most outstanding papers in the area of research, development and production of drugs from the sea, including marine natural product chemistry, that have been published in *Marine Drugs*, we would like to start an annual “Best Paper Award”. 

We are therefore pleased to announce the first “*Marine Drugs* Best Paper Award” for 2013. Nominations were selected by the Editor-in-Chief and Associate Editors of *Marine Drugs* from all papers published in 2009; reviews and articles being evaluated separately. The following three papers have won this award: 

## Article Award:

### *First* *Prize*

Jason C. Kwan, Kanchan Taori, Valerie J. Paul and Hendrik Luesch *

Lyngbyastatins 8–10, Elastase Inhibitors with Cyclic Depsipeptide Scaffolds Isolated from the Marine Cyanobacterium *Lyngbya semiplena*

*Mar. Drugs* **2009**, *7*(4), 528-538; doi:10.3390/md7040528

Available online: http://www.mdpi.com/1660-3397/7/4/528

### *Second* *Prize*

Sigrid Hakvåg, Espen Fjærvik, Geir Klinkenberg, Sven Even F. Borgos, Kjell D. Josefsen, Trond E. Ellingsen and Sergey B. Zotchev *

Violacein-Producing *Collimonas* sp. from the Sea Surface Microlayer of Coastal Waters in Trøndelag, Norway 

*Mar. Drugs* **2009**, *7*(4), 576-588; doi:10.3390/md7040576

Available online: http://www.mdpi.com/1660-3397/7/4/576

## Review Award:

### *First* *Prize*

Barbara Forte, Beatrice Malgesini, Claudia Piutti, Francesca Quartieri, Alessandra Scolaro and Gianluca Papeo *

A Submarine Journey: The Pyrrole-Imidazole Alkaloids 

*Mar. Drugs* **2009**, *7*(4), 705-753; doi:10.3390/md7040705

Available online: http://www.mdpi.com/1660-3397/7/4/705

We believe these three exceptional papers are valuable contributions to *Marine Drugs* and drug discovery research. On behalf of the Prize Awarding Committee and the Editorial Board of *Marine Drugs*, we would like to congratulate these three teams for their excellent work. In recognition of their accomplishment, Dr. Hendrik Luesch and Dr. Sergey B. Zotchev will receive a prize of 600 CHF and 400 CHF, respectively, and the privilege to publish an additional paper free of charge in open access format in *Marine Drugs*, after the usual peer-review procedure. Dr. Gianluca Papeo will also be awarded the privilege of publishing an additional research paper free of charge in open access format in *Marine Drugs*, after the usual peer-review procedure. 

## *Prize* *Awarding* *Committee*

*Editor-in-Chief* 
**Prof.** **Dr. Hartmut Laatsch**
  Institute of Organic and Biomolecular Chemistry, University of Göttingen, Tammannstrasse 2, D-37077 Göttingen, Germany
  E-Mail: hlaatsc@gwdg.de
*Associate* *Editor*
**Prof. Dr. Guido Cimino**
  Istituto di Chimica Biomolecolare (CNR) Via Campi Flegrei, 84, I-80078 Pozzuoli Napoli, Italy
  E-Mail: guido.cimino@icb.cnr.it
*Associate* *Editor*
**Prof. Dr. Jordan K. Zjawiony**
  Department of Pharmacognosy, School of Pharmacy, University of Mississippi, MS 38677, USA
  E-Mail: jordan@olemiss.edu
*Associate* *Editor*
**Prof. Dr. Nobuhiro Fusetani**
  Graduate School of Fisheries Sciences, Hokkaido University, 3-1-1 Minato-cho, Hakodate 041-8611, Japan
  E-Mail: anobu@fish.hokudai.ac.jp
*Associate* *Editor*
**Prof. Dr. Alejandro M. Mayer**
  Professor of Pharmacology, Department of Pharmacology, CCOM, Midwestern University, 555 31st. Street, Downers Grove, IL 60515, USA
  E-Mail: amayer@midwestern.edu

